# Varicella zoster virus differentially alters morphology and suppresses proinflammatory cytokines in primary human spinal cord and hippocampal astrocytes

**DOI:** 10.1186/s12974-018-1360-9

**Published:** 2018-11-15

**Authors:** Andrew N. Bubak, Christina N. Como, Anna M. Blackmon, Dallas Jones, Maria A. Nagel

**Affiliations:** 1 Department of Neurology, University of Colorado School of Medicine, 4200 E. 19th Avenue, Mail Stop B182, Aurora, CO 80045 USA; 2 Department of Ophthalmology, University of Colorado School of Medicine, Aurora, CO 80045 USA

**Keywords:** Varicella zoster virus, Encephalitis, Myelopathy, Astrocytes, Cytokines, Immune cell migration, Astrocyte heterogeneity

## Abstract

**Background:**

Varicella zoster virus (VZV) is a ubiquitous alphaherpesvirus that produces varicella and zoster. VZV can infect multiple cell types in the spinal cord and brain, including astrocytes, producing myelopathy and encephalopathy. While studies of VZV-astrocyte interactions are sparse, a recent report showed that quiescent primary human spinal cord astrocytes (qHA-sps) did not appear activated morphologically during VZV infection. Since astrocytes play a critical role in host defenses during viral infections of the central nervous system, we examined the cytokine responses of qHA-sps and quiescent primary human hippocampal astrocytes (qHA-hps) to VZV infection in vitro, as well as the ability of conditioned supernatant to recruit immune cells.

**Methods:**

At 3 days post-infection, mock- and VZV-infected qHA-sps and qHA-hps were examined for morphological changes by immunofluorescence antibody assay using antibodies directed against glial fibrillary acidic protein and VZV. Conditioned supernatants were analyzed for proinflammatory cytokines [interleukin (IL)-1β, IL-2, IL-4, IL-6, IL-8, IL-10, IL-12p70, IL-13, interferon-gamma, and tumor necrosis factor-α] using the Meso Scale Discovery multiplex ELISA platform. Finally, the ability of conditioned supernatants to attract peripheral blood mononuclear cells (PBMCs) was determined using a chemotaxis assay. Quiescent primary human perineurial cells (qHPNCs) served as a control for VZV-induced cytokine production and PBMC migration. To confirm that the astrocytes have the ability to increase cytokine secretion, qHA-sps and qHA-hps were treated with IL-1β and examined for morphological changes and IL-6 secretion.

**Results:**

VZV-infected qHA-sps displayed extensive cellular processes, whereas VZV-infected qHA-hps became swollen and clustered together. Astrocytes had the capacity to secrete IL-6 in response to IL-1β. Compared to mock-infected cells, VZV-infected qHA-sps showed significantly reduced secretion of IL-2, IL-4, IL-6, IL-12p70, and IL-13, while VZV-infected qHA-hps showed significantly reduced IL-8 secretion. In contrast, levels of all 10 cytokines examined were significantly increased in VZV-infected qHPNCs. Consistent with these results, conditioned supernatant from VZV-infected qHPNCs, but not that from VZV-infected qHA-sps and qHA-hps, recruited PBMCs.

**Conclusions:**

VZV-infected qHA-sps and qHA-hps have distinct morphological alterations and patterns of proinflammatory cytokine suppression that could contribute to ineffective viral clearance in VZV myelopathy and encephalopathy, respectively.

## Background

Varicella zoster virus (VZV) is a ubiquitous, neurotropic DNA alphaherpesvirus that produces varicella (chickenpox) upon primary infection and establishes latency in ganglionic neurons of cranial nerve, dorsal root, and autonomic ganglia, as well as adrenal gland cells [1–4]. With immunosuppression due to aging, cancer, acquired immunodeficiency syndrome, or drugs, VZV reactivates and typically spreads along nerve fibers peripherally to the skin, causing herpes zoster (shingles). VZV can also spread centrally and produce VZV myelopathy and encephalopathy, with or without the associated zoster rash. The course of these two diseases can be severe and protracted, with persistent virus infection and recurrences [5]. The mechanism(s) by which VZV evades immune clearance in the central nervous system (CNS) are not well-characterized.

The interactions of VZV with spinal cord and cortical astrocytes play an important role in the pathogenesis of VZV myelopathy and encephalopathy, respectively, since astrocytes have essential functions related to the maintenance of neural circuits including ion homeostasis, neurotransmitter clearance, synapse formation/removal, and neurovascular coupling; astrocyte dysfunction is involved in numerous CNS disorders [6, 7]. Astrocytes are also critical regulators of neuroinflammation based on their ability to regulate blood-brain barrier (BBB) permeability and infiltration of leukocytes from the circulation to the brain parenchyma [8]. These effects can be either proinflammatory through the release of molecules that disrupt the BBB, promoting extravasation of leukocytes, or anti-inflammatory through the release of molecules that promote BBB repair and suppress inflammation. Thus, astrocytes play a critical role in host defenses during viral infections.

Our knowledge of VZV-astrocyte interactions has been limited because VZV is an exclusively human virus. Postmortem immunohistochemical analyses of eight patients with VZV myelopathy demonstrated that astrocytes, as well as oligodendrocytes and neurons, in the spinal cord are infected horizontally and longitudinally [9]; postmortem immunohistochemical analyses of brains from VZV encephalitis cases showed that cortical astrocytes are preferentially infected [10–12]. In vitro studies have shown that astrocytes isolated from the human brain are permissive to VZV infection [13] and have decreased expression of glial fibrillary acidic protein (GFAP) [14]. Finally, a recent study of primary human spinal cord astrocytes revealed VZV induction of lamellipodia and filopodia that promotes viral spread in association with aberrant nuclear localization of the neurokinin-1 receptor [15]. In that study, a striking finding was the absence of morphological alterations indicative of astrocyte activation in the uninfected bystander astrocytes, suggesting the lack of “danger signals” associated with astrogliosis, cytokine production, and subsequent recruitment of inflammatory cells and microglia for viral clearance. In contrast, VZV infection of multiple other cell types, including primary human perineurial cells, fetal lung fibroblasts, brain vascular adventitial fibroblasts, and vascular smooth muscle cells, elicits secretion of multiple proinflammatory cytokines [16].

Thus, we hypothesized that unlike other VZV-infected cells, VZV-infected primary human spinal cord astrocytes do not secrete proinflammatory cytokines characteristic of astrocyte activation and are unable to recruit inflammatory cells which may contribute, in part, to persistent and recurrent VZV myelopathy. To test this hypothesis, we mock- and VZV-infected primary human spinal cord astrocytes and compared the levels of proinflammatory cytokines in the conditioned supernatant and the ability of the conditioned supernatant to attract peripheral blood mononuclear cells (PBMCs) in a chemotaxis assay. Since astrocytes from different anatomical locations have unique transcriptional profiles and responses to stimuli, we extended this study to include hippocampal astrocytes in the context of VZV encephalopathy.

## Methods

### Cells and viruses

The VZV Gilden strain, isolated from the zoster vesicle of a 75-year-old male, was propagated in human fetal lung fibroblasts (ATCC, Manassas, VA, USA) and cryopreserved at passage 3. Whole-genome-based genotyping identified the strain as a Clade 3 isolate (GenBank accession number MH379685).

Primary human spinal cord astrocytes (HA-sps; ScienCell, Carlsbad, CA, USA) and hippocampal astrocytes (HA-hps; ScienCell) were used at passage 3. Cell type was confirmed by immunofluorescence antibody assay (IFA) using an anti-GFAP antibody and 4′,6-diamidino-2-phenylindole (DAPI) nuclear DNA stain then quantitated by Fiji image processing software (https://fiji.sc/).

Cell-associated infections were used in all experiments to more accurately represent infection in vivo as described [15]. HA-sps and HA-hps were seeded at 5000 cells/cm^2^ in basal astrocyte medium containing 2% fetal bovine serum (FBS), 1% astrocyte growth supplement, and 1% 100× penicillin-streptomycin (ScienCell). After 24 h, medium was changed to basal astrocyte medium containing 0.1% FBS and 1% 100× penicillin-streptomycin and replenished every 72 h for 7 days, establishing quiescence. Quiescent HA-sps (qHA-sps) were co-cultivated with stocks of VZV-infected HA-sps (0.008 multiplicity of infection (MOI)) or mock-infected HA-sps and analyzed at 3 days post-infection (DPI) as described [15]; parallel experiments using quiescent HA-hps (qHA-hps) were also completed at the same MOI. This same protocol was followed for culturing and infecting primary human perineurial cells (HPNCs; ScienCell), which comprise the nerve-extrafascicular barrier.

While VZV is highly cell-associated and cell-free virus has not been reproducibly obtained from any primary human cell type in vitro, conditioned supernatants from each cell type were applied to uninfected HPNCs for 3 days to test for live virus. VZV DNA was not detected by qRT-PCR in any sample (data not shown) indicating that cell-free, infectious viral particles were not present in the conditioned supernatant.

Frozen human PBMCs (Stemcell Technologies, Vancouver, BC) were thawed and cultured in RPMI (ThermoFisher, Waltham, MA) supplemented with 10% human serum (Gemini Bio-Products, West Sacramento, CA) and 1% PS (Sciencell) and immediately used for migration assays.

### Immunofluorescence antibody assay

HA-sps, HA-hps, and HPNCs were plated in 24-well clear bottom plates (Ibidi, Martinsried, Germany) and cultured and infected as above. At 3 DPI, cells were washed once with 1× phosphate-buffered saline (PBS), fixed for 20 min in 4% paraformaldehyde, permeabilized with 0.3% Triton-X for 20 min, and blocked in 10% normal donkey serum. Cells were incubated with mouse anti-human VZV glycoprotein B (VZV gB; 1:500 dilution; Abcam, Cambridge, MA, USA) and chicken anti-GFAP (1:500 dilution; Abcam) antibodies overnight at 4 °C and probed with donkey anti-mouse Alexa Fluor 594 IgG (1:500 dilution; LifeTechnologies; Grand Island, NY, USA) and donkey anti-chicken Alexa Fluor 488 (1:500 dilution; Jackson ImmunoResearch Laboratories Inc.; West Grove, PA, USA). Cell nuclei were stained with 2 μg/mL DAPI. Cells were visualized by confocal microscopy (3I Marianas inverted spinning disk on Zeiss Axio observer Z1; Oberkochen, Germany) and analyzed using 3I Slidebook 6 software.

### Multiplex electrochemiluminescence immunoassay

Conditioned supernatant from mock- and VZV-infected qHA-sps, qHA-hps, and quiescent HPNCs (qHPNCs) at 3 DPI were analyzed for levels of proinflammatory cytokines using the Meso Scale Discovery Proinflammatory Panel 1 enzyme-linked immunosorbent assay (ELISA) (Meso Scale Discovery, Rockville, MD, USA) for detection of interleukin (IL)-1β, IL-2, IL-4, IL-6, IL-8, IL-10, IL-12p70, IL-13, interferon (IFN)-γ, and tumor necrosis factor (TNF)-α. Cytokine concentrations were calculated by reference to a standard curve for each molecule derived using various concentrations of the standards assayed in the same manner as supernatant samples. The lower and upper limits of detection were calculated based on the concentration of signal equal to 2.5 SDs above the zero calibrator and below the upper plateau of the standards curve, respectively. All samples were analyzed in duplicate.

### Immune cell migration assay

At 3 DPI, the ability of conditioned supernatant from VZV-infected qHA-sps, qHA-hps, and qHPNCs to recruit immune cells was determined. PBMC migration towards mock- or VZV-infected supernatant from each of the three cell lines was quantified using a chemotaxis 96-well plate with a 5-μm pore size filter (Neuroprobe, Gaithersburg, MD, USA). PBMCs were incubated with 10 μM fluorescently labeled 647-nm CellTracker (Thermo Fisher Scientific, Waltham, MA, USA) for 30 min. Sample supernatant was loaded in the wells of the 96-well chemotaxis plate according to the manufacturer’s instructions and the filter was placed on top. Labeled immune cells were pipetted onto the filter over the sample supernatant (50,000 cells/well) and incubated at 37 °C for 4 h, when the filter was removed and immune cells that migrated through the filter to the supernatant were visualized by fluorescence microscopy and quantified using Fiji 3D Objects Counter.

### IL-1β treatment of astrocytes and IL-6 quantification

To determine reactivity of HA-sps and HA-hps, quiescent cells were treated with 10 ng/mL of recombinant human IL-1β (R&D Systems, Minneapolis, MN, USA) or vehicle PBS containing 0.01% bovine serum albumin then IL-6 was quantified as previously described [17]. After 24 h, supernatant was collected, flash-frozen, and analyzed for IL-6 concentrations using the Human IL-6 ELISA MAX Deluxe set as per the manufacturer’s instructions (Biolegend, San Diego, CA, USA). In addition, cells were fixed and analyzed at 24 h post-treatment by IFA for GFAP expression and morphological changes.

### RNA extraction and quantitative PCR (qPCR)

RNA was extracted using the Direct-zol RNA MiniPrep kit (Zymo Research, Irvine) and reverse transcribed using the Transcriptor High Fidelity cDNA Synthesis Kit (Roche, Basel, Switzerland). Complementary DNA were analyzed with qPCR primers corresponding to each cytokine (IL-1β, Hs.PT.58.1518186; IL-2, Hs.PT.58.1142676; IL-4, Hs.PT.58.46539563g; IL-6, Hs.PT.58.40226675; IL-8, Hs.PT.58.39926886g; IL-10, Hs.PT.58.2807216; IL-12p70, Hs.PT.58.1687020; IL-13, Hs.PT.58.40619905.g; IFN-γ, Hs.PT.58.3781960; and TNF-α, Hs.PT.58.45380900; Integrated DNA Technologies, Coralville, IA) and glyceraldehyde-3-phosphate-dehydrogenase (GAPdH) as previously described [15]. Data were normalized to GAPdH and analyzed using the ∆∆ threshold cycle method.

### Statistical analysis

Statistical analysis was performed using GraphPad Prism (GraphPad, San Diego, CA, USA). Individual unpaired *t* tests were used to determine differences between IL-6 concentrations following IL-1β treatment. Differences in cytokine concentrations among mock- and VZV-infected qHA-sps, qHA-hps, and qHPNCs were determined using multiple unpaired *t* tests with a false discovery rate (*q* value ≤ 0.05) and the two-stage step-up method of Benjamini et al. [18]. Significant differences in number of migrated PBMCs were determined using one-way ANOVA with Tukey’s multiple comparisons test. Alpha was set at 0.05 (*p < 0.05; **p < 0.01; ***p < 0.001).

## Results

### Morphological changes differ in VZV-infected primary human spinal cord astrocytes compared to VZV-infected hippocampal astrocytes

**Fig. 1:**
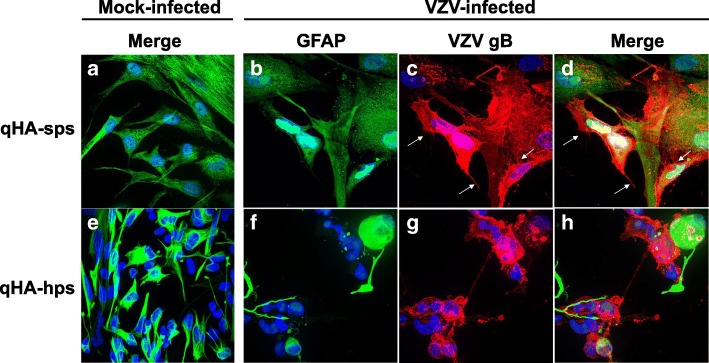
Mock and VZV infection of quiescent primary human spinal cord (qHA-sps) and hippocampal (qHA-hps) astrocytes. At 3 days post-infection, mock- and VZV-infected qHA-sps and qHA-hps were analyzed by immunofluorescent antibody assay using chicken anti-GFAP and mouse anti-VZV glycoprotein B (gB) antibodies. No mock-infected cells expressed VZV gB (**a**, **e**). In mock-infected qHA-sp cultures (**a**), all cells staining for nuclear DAPI also expressed GFAP, indicating a homogenous astrocyte culture (1208 cells counted); in mock-infected qHA-hp cultures (**e**), 99.4% of cells expressed GFAP (953 cells counted). VZV-infected qHA-sps expressed GFAP (**b**, **d**, green) and VZV gB (**c**, **d**, red); note the fine cytoskeletal filipodia-like projections sprouting from the cellular processes of virus-infected cells (**c**, **d**, arrows). VZV-infected qHA-hps expressed GFAP (**f**, **h**, green) and VZV gB (**g**, **h**, red); note the lack of filipodia (**g** and **h** compared to **c** and **d**, respectively). In addition, the VZV-infected qHA-hps appeared globular (**g**, **h**, red) compared to mock-infected qHA-hps (**e**). The blue color indicates cell nuclei. Mag × 400

GFAP was expressed in all DAPI-positive cells in mock-infected qHA-sp cultures (1208 counted) and in 99% of cells in qHA-hps (953 counted) cultures, indicating predominantly pure astrocyte cultures (Fig. 1a, e, respectively, green); in addition, qHA-sps were larger than qHA-hps (Fig. 1a, e, respectively). VZV-infected qHA-sps expressed GFAP (Fig. 1b, d, green), as well as VZV gB (Fig. 1c, d, red); note the VZV gB-positive, filipodia-like projections (arrows) sprouting from infected qHA-sps. VZV-infected qHA-hps also expressed GFAP (Fig. 1f, h) and VZV gB (Fig. 1g, h). Unlike VZV-infected qHA-sps, VZV-infected qHA-hps did not have an extensive interconnected cellular network and no VZV gB-positive, filopodia-like projections were seen. Compared to the fibroblast-like morphology of mock-infected qHA-hps (Fig. 1e), VZV-infected qHA-hps appeared swollen (Fig. 1g, h).

**Fig. 2:**
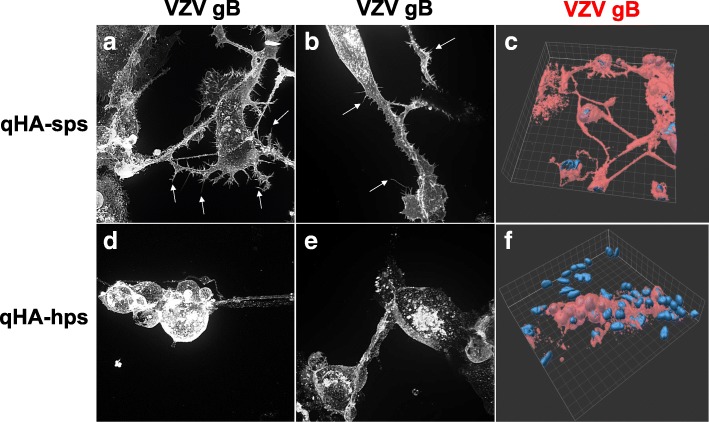
Imaging of VZV-infected quiescent primary human spinal cord (qHA-sps) and hippocampal (qHA-hps) astrocytes. At 3 days post-infection, VZV-infected qHA-sps were analyzed by immunofluorescent antibody assay using a mouse anti-VZV glycoprotein B (gB) antibody. Cells expressing VZV gB were identified and examined by enhanced contrast imaging. VZV-infected qHA-sps developed complex cellular processes with filipodia-like projections (**a**, **b**, arrows), whereas VZV-infected qHA-hps lacked the complex cellular processes and appeared globular (**d**, **e**). Surface plot imaging of VZV-infected qHA-sps demonstrated the network of cellular projections between cells (**c**), whereas VZV-infected qHA-hps formed islands of swollen, globular cells (**f**). The blue color indicates cell nuclei. Mag × 630 (**a**, **b**, **d**, and **e**) and × 400 (**c**, **f**)

Enhanced contrast imaging of VZV-infected qHA-sps (Fig. 2a, b) revealed extensive morphological changes, with abundant VZV gB-positive, filipodia-like projection sprouting along cellular processes (arrows); surface plot imaging illustrated the net-like VZV gB-positive (red) projections that link neighboring cells (Fig. 2c). The same analysis of VZV-infected qHA-hps showed distinct morphological differences, with swollen, globular cells (Fig. 2d, e) compared to the fibroblast-like structure of mock-infected qHA-hps (Fig. 1e) and without the cytoplasmic extensions seen in VZV-infected qHA-sps (Fig. 2a, b). Surface plot imaging further demonstrated the island of globular VZV-infected qHA-hps (Fig. 2f).

### Primary human spinal cord and hippocampal astrocytes can undergo morphological changes and secrete IL-6 in response to IL-1β

**Fig. 3:**
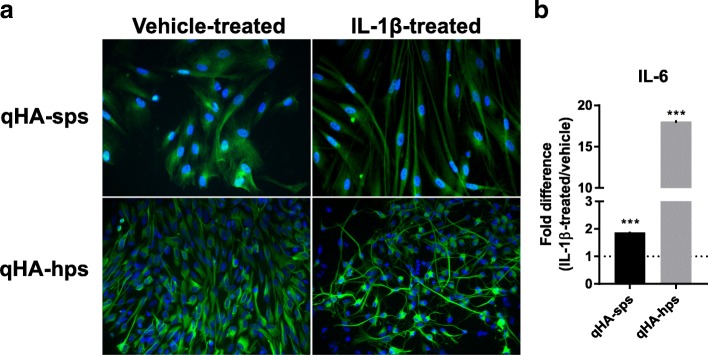
Activation signs of primary human spinal cord (qHA-sps) and hippocampal (qHA-hps) astrocytes. Compared to vehicle-treated cells (**a**, column 1), IL-1β-treated qHA-sps and qHA-hps exhibited morphological changes, with cell process extensions seen on immunofluorescence assay using anti-GFAP antibodies (**a**, column 2) at 24 h post-treatment. Compared to conditioned supernatant from vehicle-treated cells, IL-1β-treated qHA-sps and qHA-hps showed significantly increased IL-6 (1.87 ± 0.02; 18.04 ± 0.14, respectively; mean fold change ± SD; *p* < 0.001, *n* = 3) (**b**). The morphological changes in both astrocyte types and the increased secretion of IL-6 in response to IL-1β stimuli are consistent with astrocyte activation. The blue color indicates cell nuclei. Mag × 400

At 24 h after treatment of qHA-sps and qHA-hps with 10 ng/mL of IL-1β, both cell types exhibited morphological changes, with cell process extensions seen on IFA using anti-GFAP antibodies, as compared to vehicle-treated cells (Fig. 3a). IL-1β-treated qHA-sps (*n* = 3) and qHA-hps (*n* = 3) had significantly increased concentrations of IL-6 (416.77 ± 69.6 versus 780.98 ± 8.48, *p* < 0.001; 42.21 ± 20.24 versus 761.73 ± 5.87, *p* < 0.001; respectively; mean pg/mL ± SD), i.e., a fold change of 1.87 ± 0.02 and 18.04 ± 0.14, respectively (mean fold change ± SD), as compared to the supernatant from vehicle-treated cells (Fig. 3b). The morphological changes in both astrocyte types, as well as increased secretion of IL-6 in response to IL-1β stimuli, provide evidence of the capability of these cells to respond to exogenous factors and increase cytokine production/release.

### Unlike the proinflammatory environment produced by VZV-infected primary human perineurial cells, neither proinflammatory cytokines nor immune cell migration is significantly increased in VZV-infected primary human spinal cord and hippocampal astrocytes

**Fig. 4:**
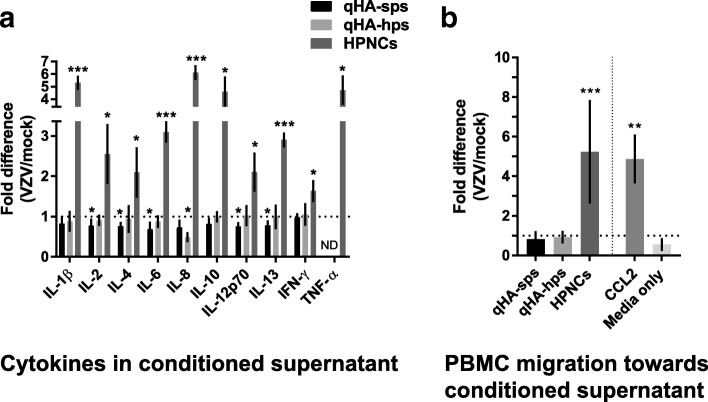
Cytokines in VZV-infected quiescent primary human spinal cord (qHA-sps) and hippocampal (qHA-hps) astrocyte conditioned supernatants. At 3 days post-infection, supernatants from mock- and VZV-infected cells were analyzed for cytokines (IL-1β, IL-2, IL-4, IL-6, IL-8, IL-10, IL-12p70, IL-13, IFN-γ, and TNF-α) by Meso Scale Discovery multiplex assays (**a**). VZV/mock-infected qHA-sps (black bars) showed significant fold changes in IL-2 (0.74 ± 0.17), IL-4 (0.70 ± 0.1), IL-6 (0.60 ± 0.17), IL-12p70 (0.70 ± 0.1), and IL-13 (0.73 ± 0.11), while VZV/mock-infected qHA-sps (light gray bars) had only significantly reduced IL-8 levels (fold change 0.49 ± 0.12). VZV/mock-infected quiescent human primary perineurial cells (qHPNCs, dark gray bars) showed significant fold changes in IL-1β (5.31 ± 0.56), IL-2 (2.55 ± 0.74), IL-4 (2.09 ± 0.62), IL-6 (3.09 ± 0.27), IL-8 (6.11 ± 0.59), IL-10 (4.59 ± 1.21), IL-12p70 (2.1 ± 0.48), IL-13 (2.9 ± 0.18), IFN-γ (1.63 ± 0.27), and TNF-α (4.71 ± 1.17). Bar graphs represent mean ± SD fold change in cytokine levels normalized to respective mock groups (dashed line represents 1 or no change; *n* = 3). (**b**). Chemotaxis analysis to test the functional relevance of cytokines produced by VZV-infected qHA-sps or qHA-hps revealed no significant differences in the number of migrated peripheral blood mononuclear cells (PBMCs) as compared to that in their respective mock-infected conditioned supernatants. Positive controls VZV-infected HPNCs and chemokine (C-C motif) ligand 2 (CCL-2) diluted in quiescent astrocyte medium (20 pg/mL) showed a significant increase in migrated PBMCs (5.24 ± 2.61 and 4.88 ± 1.22 respectively; *n* = 2). The negative control quiescent astrocyte medium only did not attract PBMCs. Bar graphs represent mean ± SD fold change in immune cell migration numbers compared to the respective mock groups (dashed line represents 1 or no change)

At 3 DPI, compared to conditioned supernatant from mock-infected qHA-sps (*n* = 4), supernatant from VZV-infected qHA-sps (*n* = 5) had significantly reduced concentrations of IL-2 (*p* = 0.04; *q* = 0.042), IL-4 (*p* = 0.014; *q* = 0.03), IL-6 (*p* = 0.03; *q* = 0.04), IL-12p70 (*p* = 0.016; *q* = 0.03), and IL-13 (*p* = 0.01; *q* = 0.03) released into the supernatant, translating to a significant fold change of VZV/mock-infected qHA-sps for IL-2 (0.74 ± 0.17), IL-4 (0.70 ± 0.1), IL-6 (0.60 ± 0.17), IL-12p70 (0.70 ± 0.1), and IL-13 (0.73 ± 0.11) (mean ± SD) (Fig. 4a, black bars); no differences in IL-1β, IL-8, IL-10, IFN-γ, or TNF-α were detected. Compared to supernatant from mock-infected qHA-hps (*n* = 5), VZV-infected qHA-hps (*n* = 4) had only significantly reduced concentrations of IL-8 (*p* = 0.002; *q* = 0.024), with a fold change of 0.49 ± 0.12; (mean ± SD) (Fig. 4a, light gray bars). RNA transcripts were not detected for any cytokines corroborating the reduced or unchanged secreted protein levels measured in both mock- or VZV-infected qHA-sps and qHA-hps.

In contrast, qHPNCs infected with the same VZV Gilden strain displayed significantly elevated levels of all secreted cytokines measured by ELISA compared to mock-infected qHPNCs (*n* = 3): IL-1β (*p* = 0.0002; q = 0.0005), IL-2 (*p* = 0.022; *q* = 0.026), IL-4 (*p* = 0.041; *q* = 0.043), IL-6 (*p* = 0.0002; *q* = 0.0005), IL-8 (*p* = 0.0001; *q* = 0.0005), IL-10 (*p* = 0.007; *q* = 0.014), IL-12p70 (*p* = 0.018, *q* = 0.025), IL-13 (*p* < 0.0001; *q* = 0.0005), IFN-γ (*p* = 0.016; *q* = 0.025), and TNF-α (*p* = 0.005; *q* = 0.01), translating to a significant fold change of VZV/mock-infected qHPNCs for IL-1β (5.31 ± 0.56), IL-2 (2.55 ± 0.74), IL-4 (2.09 ± 0.62), IL-6 (3.09 ± 0.27), IL-8 (6.11 ± 0.59), IL-10 (4.59 ± 1.21), IL-12p70 (2.1 ± 0.48), IL-13 (2.9 ± 0.18), IFN-γ (1.63 ± 0.27), and TNF-α (4.71 ± 1.17) (mean ± SD; Fig. 4a, dark gray bars). The ability of the VZV Gilden strain to induce secretion of proinflammatory cytokines in HPNCs is consistent with results using the VZV Ellen strain in a prior report [16]. Transcripts for IL-10 were not detected in either mock- or VZV-infected qHPNCs. RNA transcripts for IL-2 and IFN-γ were not detected in mock-infected qHPNCs but were detected in all VZV-infected qHPNCs (17.5 ± 1.16 and 16.78 ± 0.71, respectively; *n* = 3, delta CT ± SD relative to GAPDH). Compared to mock-infected qHPNCs, transcripts in VZV-infected qHPNCs showed an upward trend for IL-4 (1.56 ± 0.29), IL-8 (6.7 ± 5.42), IL-13 (19.7 ± 18.21), and TNF-α (53.68 ± 18.31) but a downward trend for IL-1β (0.19 ± 0.11) and IL-12p70 (0.05 ± 0.03) (mean fold change ± SD; *n* = 3). IL-6 transcripts remained unchanged (0.98 ± 0.05) (mean fold change ± SD; *n* = 3). The discrepancy between secreted cytokine increases in VZV-infected cells and the undetectable, unchanged, or decreased transcripts for IL-10, IL-1β, IL-12p70, and IL-6 may be due to a negative feedback loop, transcripts below the level of quantitation on qPCR, or the fact that secreted cytokines represented cumulative cytokine production over the 3 days of infection, whereas transcripts only represented a single point.

To determine the functional significance of cytokines detected, the ability of conditioned supernatant to attract PBMCs was measured in a chemotaxis assay. Consistent with the reduced or unchanged cytokine concentrations, the number of migrating PBMCs towards supernatant from either VZV-infected qHA-sps or qHA-hps did not differ from that in their respective mock groups (0.82 ± 0.41 and 0.92 ± 0.32, respectively; mean fold change ± SD; *p* > 0.05, *n* = 7) (Fig. 4b, black and light gray bars, respectively). In contrast, conditioned supernatant from VZV-infected qHPNCs, which had elevated levels of multiple proinflammatory cytokines, attracted significantly more PBMCs compared to mock-infected qHPNCs (5.24 ± 2.61; mean fold change ± SD; *p* < 0.001, *n* = 4) (Fig. 4b, dark gray bars, respectively). Chemokine (C-C motif) ligand 2 (CCL-2), used as a positive control (20 pg/mL, diluted in quiescent astrocyte media), attracted a significant number of PBMCs (4.88 ± 1.22, mean fold change ± SD relative to medium only, *p* < 0.01, *n* = 2); quiescent astrocyte medium (0.56 ± 0.19, *n* = 3; mean fold change ± SD) was used as a negative control (Fig. 4b).

## Discussion

Herein, we found that VZV infection of primary human spinal cord and hippocampal astrocytes does not attract PBMCs or induce the release of proinflammatory cytokines; indeed, VZV infection actually suppressed basal levels of many cytokines, with suppressed levels of secreted IL-2, IL-4, IL-6, IL-12p70, and IL-13 in VZV-infected qHA-sps and suppressed levels of secreted IL-8 in VZV-infected qHA-hps as compared to their mock-infected counterparts. The inability of VZV to induce release of proinflammatory cytokines in these two primary human astrocyte cell lines was not due to the VZV strain since this same strain elicited a robust proinflammatory response in HPNCs or due to the inability of the qHA-sps and qHA-hps to mount an inflammatory response since addition of IL-1β produced morphological changes and subsequent secretion of IL-6 in both cell types consistent with other studies [17]. Rather, these in vitro observations reflect a unique VZV-astrocyte interaction that may contribute to ineffective immune clearance of VZV-infected spinal cord and hippocampal astrocytes in the context of VZV myelopathy and encephalopathy, respectively.

While there have been several studies of astrocyte responses to viral infections in animal models, human astrocytes differ significantly from mouse or rat astrocytes in gene expression, function, and responses to pathogens, as well as in responses to glutamate [19] and in toll-like receptor expression and signaling [20, 21]. Thus, we compared our findings to other viral studies that used human astrocytes exclusively. Herpes simplex virus type 1 (HSV-1), another alphaherpesvirus, can also productively infect human fetal cortical astrocytes without production of cytokines; however, HSV-1 infection of microglia in the same study led to upregulation of TNF-α, IL-1β, IP-10, and RANTES, along with lower amounts of IL-6, IL-8, and macrophage inflammatory protein (MIP)-1α [22], indicating cell type-specific responses to virus infection among CNS cells. Furthermore, treatment of HSV-1-infected astrocytes with IFN-γ led to upregulation of endogenous IFN-α/β and several IFN-stimulated genes and cytokines that suppressed HSV-1 replication [23].

Unlike the lack of proinflammatory cytokine induction during VZV and HSV-1 infection of human astrocytes, most other viruses do, in fact, elicit the release of a spectrum of proinflammatory cytokines that contribute to neuroinflammation and associated disease. During infection of primary human cortical astrocytes, for example, cytomegalovirus induces release of chemoattractant protein-1 and IL-8 [24], while human immunodeficiency virus (HIV) induces release of IL-6 [25] and Zika virus induces release of CXCL-10, IL-6, IL-8, IL-12, and RANTES [26]. Moreover, infection of primary human cortical astrocytes by tick-borne encephalitis virus results in a persistent, productive infection that is associated with astrocyte activation and upregulation of several proinflammatory cytokines/chemokines including IFN-γ, TNF-α, IL-1β, IL-6, IL-8, IFN-γ-induced protein 10, MIP, and RANTES [27, 28]. Enterovirus 71 infection of human astrocytes from the cerebrum, brain stem, and hippocampus increases secretion of IL-6 and IL-8 [29]. In coxsackievirus B3-infected human progenitor-derived astrocytes, the proinflammatory cytokine vascular cell adhesion molecule 1, IL-6, and IL-8 are upregulated [30]. In a U251 astrocyte cell line derived from human glioblastoma astrocytoma, H5N1 influenza virus infection increases expression of multiple proinflammatory cytokines, including IL-6, IL-8, and TNF-α, in microarray analysis and by qRT-PCR [31], and Japanese encephalitis virus infection increases IFN-γ, TNF-α, IL-1β, IL-6, and chemokine (C-C motif) ligand 5 [32].

A notable finding in the present study is the distinct morphology and specific cytokines suppressed in VZV-infected qHA-sps compared to VZV-infected qHA-hps. Mock-infected qHA-sps were larger than qHA-hps and had higher basal levels of all ten cytokines examined, with about 10- and 100-fold higher basal levels of IL-6 and IL-8, respectively, in mock-infected qHA-sps. Interestingly, the fold increase in IL-6 secretion following IL-1β treatment was substantially higher in qHA-hps than qHA-sps. After VZV infection, qHA-sps produced a network of cellular processes that extended to other cells, whereas qHA-hps appeared swollen and formed isolated clusters of virus-infected islands without cellular projections. These differences between qHA-sps and qHA-hps are consistent with the emerging body of research describing astrocyte heterogeneity (reviewed in ref. [7]) at the transcriptional and functional levels, wherein astrocytes from different regions of the CNS have been shown to have distinct and unique functions within non-overlapping domains. These unique functional subtypes can be observed within microcircuits as well as in distant anatomical locations, as exemplified by a study comparing dorsal horn and ventral horn astrocytes, which identified differential gene expression profiles critical to the integrity of motor neuron circuits [33], and by studies showing that astrocytes in the CA1 or CA3 region of the hippocampus display diverse Ca^2+^ signaling properties in response to minimal or intense stimulation, with important implications for local versus neuronal network synchronization [7, 34, 35].

Finally, the lack of robust proinflammatory cytokine secretion from VZV-infected astrocytes in these in vitro studies is discordant with what is seen clinically in VZV myelopathy. In a study of 10 patients with virologically verified VZV myelopathy, laboratory and imaging findings support the notion of an inflammatory process in the majority of cases with nine of ten patients having elevated leukocytes in their cerebrospinal fluid (CSF), enhancing spinal lesions on magnetic resonance imaging or both [36]. While the cytokine profile of CSF from VZV myelopathy patients has not been extensively characterized, in another central nervous system disease produced by VZV (VZV vasculopathy), CSF contains increased levels of IL-6 and IL-8 compared to CSF from normal patients and those with multiple sclerosis [37]. Thus, our findings indicate that other cell types may contribute to the proinflammatory environment seen in the spinal cord and CSF of patients with VZV myelopathy.

## Conclusions

The absence of human spinal cord and hippocampal astrocyte activation, proinflammatory cytokine release, and recruitment of immune cells during VZV infection may represent an immune evasion strategy during VZV myelopathy and encephalopathy. These findings raise important questions about the mechanisms by which VZV infection suppresses astrocyte activation, as well as the role of VZV-infected astrocytes in the in vivo microenvironment where other cell types, such as microglia, are involved and inflammation is detected in association with clinical disease.

## Abbreviations

BBB: Blood-brain barrier
CCL-2: Chemokine (C-C motif) ligand 2
CNS: Central nervous system
DAPI: 4′,6-Diamidino-2-phenylindole
DPI: Days post-infection
ELISA: Enzyme-linked immunosorbent assay
FBS: Fetal bovine serum
gB: Glycoprotein B
GFAP: Glial fibrillary acidic protein
HA-hps: Primary human hippocampal astrocytes
HA-sps: Primary human spinal cord astrocytes
HIV: Human immunodeficiency virus
HPNCs: Primary human perineurial cells
HSV-1: Herpes simplex virus type 1
IFA: Immunofluorescence antibody assay
IFN-γ: Interferon gamma
IL: Interleukin
MOI: Multiplicity of infection
PBMC: Peripheral blood mononuclear cell
PBS: Phosphate-buffered saline
qHA-hps: Quiescent primary human hippocampal astrocytes
qHA-sps: Quiescent primary human spinal cord astrocytes
qHPNCs: Quiescent primary human perineurial cells
TNF-α: Tumor necrosis factor alpha
VZV: Varicella zoster virus
